# Preterm birth and PM_2.5_ in Puerto Rico: evidence from the PROTECT birth cohort

**DOI:** 10.1186/s12940-021-00748-5

**Published:** 2021-06-11

**Authors:** Kipruto Kirwa, Zlatan Feric, Justin Manjourides, Akram Alshawabekeh, Carmen Milagros Velez Vega, José F. Cordero, John D. Meeker, Helen H. Suh

**Affiliations:** 1 Department of Environmental and Occupational Health Sciences, University of Washington, Seattle, WA USA; 2 Department of Civil and Environmental Engineering, Northeastern University, Boston, MA USA; 3 Department of Health Sciences, Northeastern University, Boston, MA USA; 4 Department of Social Sciences, University of Puerto Rico, San Juan, Puerto Rico; 5 Department of Epidemiology and Biostatistics, University of Georgia, Athens, GA USA; 6 School of Public Health, University of Michigan, Ann Arbor, MI USA; 7 Department of Civil and Environmental Engineering, Tufts University, Anderson Hall, 200 College Avenue, Medford, MA 02155 USA

**Keywords:** PM_2.5_, Preterm birth, Adverse birth outcomes, Prenatal exposure, Puerto Rico

## Abstract

**Background:**

Preterm birth (PTB, birth before 37 weeks of gestation) has been associated with adverse health outcomes across the lifespan. Evidence on the association between PTB and prenatal exposure to air pollutants is inconsistent, and is especially lacking for ethnic/racial minority populations.

**Methods:**

We obtained data on maternal characteristics and behaviors and PTB and other birth outcomes for women participating in the Puerto Rico Testsite for Exploring Contamination Threats (PROTECT) cohort, who lived in municipalities located along the North Coast of Puerto Rico. We assessed pre-natal PM_2.5_ exposures for each infant based on the nearest US Environmental Protection Agency monitor. We estimated prenatal phthalate exposures as the geometric mean of urinary measurements obtained during pregnancy. We then examined the association between PM_2.5_ and PTB using modified Poisson regression and assessed modification of the association by phthalate exposure levels and sociodemographic factors such as maternal age and infant gender.

**Results:**

Among 1092 singleton births, 9.1% of infants were born preterm and 92.9% of mothers had at least a high school education. Mothers had a mean (standard deviation) age of 26.9 (5.5) years and a median (range) of 2.0 (1.0–8.0) pregnancies. Nearly all women were Hispanic white, black, or mixed race. Median (range) prenatal PM_2.5_ concentrations were 6.0 (3.1–19.8) *μ* g/m^3^. Median (interquartile range) prenatal phthalate levels were 14.9 (8.9–26.0) and 14.5 (8.4–26.0), respectively, for di-n-butyl phthalate (DBP) and di-isobutyl phthalate (DiBP). An interquartile range increase in PM_2.5_ was associated with a 1.2% (95% CI 0.4, 2.1%) higher risk of PTB. There was little difference in PTB risk in strata of infant sex, mother’s age, family income, history of adverse birth outcome, parity, and pre-pregnancy body mass index. Pregnancy urinary phthalate metabolite levels did not modify the PM_2.5_-PTB association.

**Conclusion:**

Among ethnic minority women in Puerto Rico, prenatal PM_2.5_ exposure is associated with a small but significant increase in risk of PTB.

## Introduction

Preterm birth (PTB) is the largest cause of infant death in the United States, and has been associated with morbidity and mortality throughout the lifespan [1–3]. Post-neonatal morbidity due to jaundice, impaired kidney function, infectious agents, and respiratory distress is higher among prematurely-born infants [4]. Longer term, children born prematurely are more likely to exhibit psycho-behavioral, developmental, and neurocognitive deficits, higher incidence of asthma and chronic lung diseases, and are more predisposed to hearing loss, dental problems, retinopathy, cerebral palsy, cardiovascular disorders, gastrointestinal diseases and infections affecting the lungs and brain [2, 4, 5]. In the US, where the average rate of PTB has recently increased to approximately 10%, about 36% of infant deaths are due to PTB-related causes, and annual costs associated with PTB exceed $26 billion [1, 6, 7]. PTB occurred 50% more frequently among black than white infants in 2018; and relative to 2017, incidence increased significantly more among black and Hispanic mothers compared to their white counterparts [7]. Given its burden, skewed ethnic/racial risk profile, and economic and health consequences, developing a better understanding of the modifiable correlates of PTB remains essential.

Although mechanisms through which air pollutants may lead to PTB have been postulated [8–10], evidence on the association between exposure to particulate matter less than 2.5 μm in aerodynamic diameter (PM_2.5_) and PTB remains inconclusive. For a 10 *μ* g/m^3^ increase in entire-pregnancy PM_2.5_ exposure, one recent meta-analysis reported a statistically significant pooled odds ratio (1.13, 95% confidence interval [CI] 1.03, 1.24) [11], while another did not (1.02, 95% CI 0.93, 1.12), [12] suggesting the need for more evidence. In a more recent systematic review restricted to cohort studies, nine of 18 studies reported non-significant or negative associations between PTB and prenatal PM_2.5_ exposure [13].

While pollutant exposure in the US is known to be higher among racial/ethnic minority populations [14], and notwithstanding the uneven racial risk profile of PTB [15], nearly all previous studies have been conducted among predominantly white populations, and in particular, none focused on a Hispanic population residing outside the contiguous United States. The few studies that include multiple race/ethnicities suggest that the adverse effects of PM_2.5_ on birth outcomes may be stronger among non-white mothers [16]. We examined the association of prenatal PM_2.5_ exposure and PTB in a predominantly Hispanic and black cohort of Puerto Rican women, and assessed whether PTB risk varied by maternal sociodemographic factors, and infant sex. In previous work, we postulated that the elevated PTB rates in Puerto Rico may be the result of a combination of environmental exposures that jointly exploit similar pathophysiologic mechanisms on the pathway to PTB [17]. We have reported on the association between phthalates and PTB in this study population [17], and there is evidence that, like air pollutants, phthalates may operate via increased inflammation and oxidative stress [18, 19]. Therefore, we hypothesized that the impact of air pollution exposures on adverse birth outcomes may be enhanced in mothers with high phthalate exposures and assessed whether levels of phthalate exposure modified the PTB risk associated with PM_2.5_.

## Methods

### Study design and population

We identified all live singleton births that occurred between August 2011 and August 2018 to women enrolled in the ongoing Puerto Rico Testsite for Exploring Contamination Threats (PROTECT) prospective cohort study. With support from the NIEHS Superfund Research Program, the goal of the PROTECT study is to examine environmental contributors to PTB and other adverse birth outcomes in Puerto Rico, which suffers high levels of such outcomes compared to other US regions. The design of PROTECT has been described in detail previously [20, 21]. Briefly, participants were recruited from seven prenatal clinics in northern Puerto Rico at about 14 weeks of gestation and followed until birth. Mothers included in the study were aged between 18 and 40 years and did not have any major medical or obstetric complications. Up to three study visits were conducted per participant, at approximately 18, 22 (home visit), and 26 weeks of gestation. At these visits, detailed data on maternal demographic, behavioral, and pregnancy history characteristics, as well as spot urine samples, were obtained. Infant data were obtained from birth records. Ethics review boards at the University of Puerto Rico, Northeastern University, and Tufts University provided approval.

### Exposure assessment

Daily PM_2.5_ concentrations were obtained from US Environmental Protection (EPA) monitors closest to the municipalities in which mothers resided at the time of birth. The monitors, which use Federal Reference Method (FRM) filtration techniques consistent with the National Ambient Air Quality Standards (NAAQS), typically measured PM_2.5_ concentrations every third day, based on gravimetric assessment of particulates deposited on an active filter over a single 24-h periods on a one in every three-day schedule. PM_2.5_ exposures were estimated for each baby as the measured concentration at the monitor closest to the mother’s municipality of residence. If monitors were co-located, or there were more than one in a municipality, the monitor with the least missing data during the relevant prenatal periods were used. The average distance from a monitor to a participant residence was 10.6 km. These stationary ambient monitors were community monitors that followed EPA siting criteria, and were therefore located from away from major PM_2.5_ sources.

We processed pollutant concentration data as described earlier [22]. Briefly, we assessed each monitor’s data missingness pattern. When a monitor had fewer than seven PM_2.5_ values in a given month, we imputed missing daily PM_2.5_ concentrations using a random regression imputation technique [23], in which we estimated the PM_2.5_ concentration from the most appropriate monitor with non-missing data for that day. The choice of appropriate monitor was based on inter-monitor correlation between non-missing PM values, its proximity to the monitor with missing data, and the predominant wind direction. PM_2.5_ values at nearby monitoring sites were strongly correlated (Pearson correlation coefficients 0.74–0.91). We did not impute exposure values for a monitor if no appropriate monitor from which to impute was identified. We then applied a uniform smoother to each monitor’s imputed concentration series to calculate the average exposure estimate for each baby’s prenatal period. Exposure estimates were considered valid if at least 75% of the expected exposure data were available.

### Outcome

We adopted the generally accepted definition of PTB as birth before completion of 37 weeks of gestation [24]. As previously described, gestational age in this cohort was ascertained from birth records, using ultrasound estimates when available and self-reported date of last menstrual period otherwise, in accordance with best-practice recommendations from the American Congress of Gynecologists [25, 26].

### Covariates

Maternal data from study questionnaires included age (continuous), municipality of residence at the time of infant birth, number of pregnancies, education level (high school or less/more than high school), race/ethnicity (Hispanic white/black or other), history of previous abortion, stillbirth or premature birth, pre-pregnancy body mass index, marital status, employment status, annual family income, exercise habits (**≥**30/< 30 min per day in the past 3 months), type of regular means of commuting (motorized/non-motorized or none) and smoke exposure (any/no daily exposure to cigarette smoke in the home). From birth records we obtained information on infant sex and type of delivery (spontaneous vaginal or Caesarian).

Procedures for collection and processing of phthalate exposure data have been outlined in detail previously [17]. Briefly, urine samples collected at three time points during pregnancy were analyzed for phthalate metabolites at the Centers for Disease Control using online solid phase extraction high-performance liquid chromatography-isotope tandem mass spectrometry. In this study, we used geometric means of all specific gravity-adjusted measurements of across pregnancy. For analysis, we used the molar sums of metabolites of di-n-butyl phthalate (DBP) and di-isobutyl phthalate (DiBP), because they have recently been shown to be associated with preterm birth in this cohort [17]. We log-transformed metabolite concentrations to normalize their distributions and for consistency with past research.

We also obtained area-level socioeconomic and health indicators for Puerto Rican municipalities from the American Community Survey (ACS, https://www.census.gov/programs-surveys/acs), including population density, income per capita (in 2013 inflation adjusted dollars), proportion of non-white non-Hispanic residents, average unemployment rate over the period 2004–2013, proportion of households with at least one of four severe US Department of Housing and Urban Development-designated problems (overcrowding, high housing cost, lack of kitchen, lack of plumbing), percentage of residents with a less than high school level of education, and the age adjusted prevalence of diabetes mellitus over the period 2004–2013.

### Statistical analysis

We used a modified Poisson regression model with a sandwich linearized estimator of variance to estimate the association between average prenatal PM_2.5_ exposure and PTB in the PROTECT birth cohort [27]. This allowed us to obtain a direct measure of the risk ratio, which we then scaled to represent effect per interquartile range change in average PM_2.5_ exposure level.

Model covariates were selected based on documented relevance to either exposure or outcome [28, 29]. In base models, we adjusted for individual-level covariates including maternal sociodemographic characteristics, physical activity patterns, smoke exposure, history of adverse birth outcomes, and infant’s sex. Due to previous evidence suggesting importance of area-level variables for adverse birth outcomes [22], we further adjusted for municipality-level indicators of sociodemographic, housing, and diabetes prevalence characteristics. In all models we allowed for potential clustering by municipality.

We assessed the risk of PTB in strata of infant sex, mother’s age (< 25 vs > = 25 years), family income (< 30 K vs > = 30 K USD), history of adverse birth outcome, number of previous pregnancies, pre-pregnancy body mass index, and degree of prematurity (24–34 weeks vs 34–36 weeks). We also evaluated whether PTB risk varied by pregnancy phthalate levels using multiplicative interaction terms in the regression models. Analyses were performed using Stata 15.1 (StataCorp, TX) and R 3.6.0 (R Foundation for Statistical Computing, Vienna, Austria).

## Results

Our analytical dataset consisted of 1092 PROTECT participants from 21 municipalities in Puerto Rico who had live singleton births between August 2011 and August 2018. About 9.1% of births occurred preterm, and 47.8% of deliveries were Caesarian, levels which are consistent with those of the whole Puerto Rican population [30, 31]. Mothers had a mean age (standard deviation) of 26.9 (5.5) years, at least a high school level of education (92.9%), and majority identified as Hispanic white (52.5%) (Table 1). Women reported a median (range) of 2.0 (1.0–8.0) pregnancies, and 47.6% of infants were female. The proportion of mothers reporting the current pregnancy as her first was 41.8 and 39.4% for those delivering term and preterm infants, respectively. Of mothers whose infants were born pre-term, 31.3% reported a history of previous spontaneous abortion, stillbirth or premature birth, compared to 23.0% among those whose infants reached term (Table 1). The respective average gestational ages (35.1 and 34.6 weeks) and birth weights (2411 and 2421 g) among Caesarean and vaginal preterm births were similar. The distribution of small and large for gestational age infants was similar across term and preterm births. About 9.6% (*n* = 97) of term births and 9.1% of preterm births (*n* = 9) were small for gestational age. The corresponding large for gestational age proportions were 9.2% (*n* = 91) and 13.1% (*n* = 13) for term and preterm infants, respectively.

**Table 1:** Characteristics associated with live births occurring between 2011 and 2018 among participants in the PROTECT cohort^a^

Characteristic	All births (*N* = 1092)	Term births (*n* = 993)	Preterm births (*n* = 99)
Individual-level characteristics			
Infant sex			
Female	519 (47.6)	479 (48.3)	40 (40.4)
Male	572 (52.4)	513 (51.7)	59 (59.6)
Length of gestation, mean (SD), weeks	38.5 (1.8)	38.9 (1.1)	34.9 (3.2)
Mother’s age, mean (SD), years	26.9 (5.5)	26.9 (5.5)	27.0 (5.6)
Mother’s race			
Hispanic white	573 (52.5)	527 (53.1)	46 (46.5)
Black or other race	498 (45.6)	446 (44.9)	52 (52.5)
Mother’s level of education^b^			
Less than high school	76 (7.1)	64 (6.6)	12 (12.2)
High school or technical school	515 (47.9)	462 (47.3)	53 (54.1)
College	484 (45.0)	451 (46.2)	33 (33.7)
Family income in past year, US dollars			
< 10,000	270 (24.7)	231 (23.3)	37 (37.4)
10,000 – 29,999	308 (28.2)	280 (28.2)	25 (25.3)
30,000 – 49,999	227 (20.8)	211 (21.2)	15 (15.2)
≥ 50,000	138 (12.6)	125 (12.6)	11 (11.1)
Consumed alcoholic drink on one or more days per week in past year	176 (16.1)	165 (16.6)	11 (11.1)
Drinks consumed per drinking occasion, past year			
0	584 (59.8)	522 (59.0)	62 (66.7)
1–2	228 (23.3)	209 (23.6)	19 (20.4)
3 or more	165 (16.9)	153 (17.3)	12 (12.9)
Ever smoked	172 (15.9)	160 (16.3)	12 (12.2)
Current smoker	15 (1.4)	13 (1.3)	2 (2.0)
Partner smokes at home	107 (9.8)	90 (9.1)	17 (17.2)
Birth weight, mean (SD), grams	3161.9 (523.8)	3235.1 (442.5)	2415.4 (683.2)
Number of pregnancies, including this one			
1	454 (41.6)	415 (41.8)	39 (39.4)
2	392 (35.9)	362 (36.5)	30 (30.3)
3 or more	232 (21.2)	203 (20.4)	29 (29.3)
Number of other children, excluding this one			
0	414 (37.9)	377 (38.0)	37 (37.4)
1	420 (38.5)	382 (38.5)	38 (38.4)
2 or more	123 (11.3)	105 (10.6)	18 (18.2)
Has ever experienced adverse birth outcome (spontaneous abortion, preterm birth, or premature birth)	259 (23.7)	228 (23.0)	31 (31.3)
Marital status at first visit			
Single or divorced	209 (19.1)	191 (19.2)	18 (18.2)
Married	615 (56.3)	569 (57.3)	46 (46.5)
Cohabiting	255 (23.4)	221 (22.3)	34 (34.3)
Employed at time of first visit	688 (63.0)	635 (63.9)	53 (53.5)
Pre-pregnancy BMI, mean (SD)	25.2 (5.4)	25.1 (5.4)	26.6 (5.9)
Maternal pre-pregnancy BMI			
Underweight (< 18.5)	69 (6.3)	65 (6.5)	4 (4.0)
Normal (18.5–24.9)	500 (45.8)	464 (46.7)	36 (36.4)
Overweight (25–29.9)	284 (26.0)	256 (25.8)	28 (28.3)
Obese (> 30)	179 (16.4)	158 (15.9)	21 (21.2)
Delivered by Caesarian section	522 (47.8)	464 (46.7)	58 (58.6)
Exercised at least 30 min per day in past year	211 (19.3)	193 (19.4)	18 (18.2)
Has a regular motorized commute (car, bus, train)	952 (87.2)	874 (88.0)	78 (78.8)
Municipal-level characteristics^c^			
Median household income in 2013 inflation-adjusted US dollars, mean (SD)	16,285.3 (2539.5)	16,331.8 (2563.0)	15,819.5 (2249.8)
Population density per sq. mile, mean (SD)	884.0 (384.3)	890.7 (386.8)	817.4 (353.6)
% adults (> 25 yr) with < high school education, %, mean (SD)	35.4 (5.5)	35.3 (5.6)	36.4 (4.9)
% occupied units with 1 or more severe housing problems, mean (SD)^d^	26.6 (8.9)	26.7 (9.0)	25.9 (8.1)
% population that is non-white non-Hispanic, mean (SD)	11.7 (8.3)	11.6 (8.3)	12.6 (8.8)
Unemployment rate 2004–2013, %, mean (SD)	16.2 (2.3)	16.1 (2.3)	16.7 (2.4)
Mean age-adjusted diabetes prevalence 2004–2013, %, mean (SD)	14.1 (0.6)	14.1 (0.6)	14.3 (0.5)

^a^N (%) unless otherwise specified

^**b**^Education levels are equivalent to: Less than high school – did not go to secondary school; High school or technical school – started or completed secondary education and/or trade school; College – started or completed two-year tertiary education or university

^**c**^These data are publicly available at https://www.census.gov/programs-surveys/acs/data.html and https://www.huduser.gov/portal/datasets/cp.html

^d^These are designated by the US Department of Housing and Urban Development as overcrowding, lack of kitchen, lack of plumbing, and high housing cost

The average (interquartile range, IQR) concentrations of PM_2.5_ over the pregnancy duration was 6.8 (5.4–7.4) *μ* g/m^3^, with averages of 6.8 and 6.4 *μ* g/m^3^ for term and preterm births, respectively. The range of PM_2.5_ exposures observed among the study population was 3.1 to 19.8 *μ* g/m^3^. In fully-adjusted models, greater PTB risk was associated with higher maternal age, higher BMI, lower levels of municipal-level educational attainment and higher municipal-level diabetes prevalence (Table 2). Our data were also suggestive of higher PTB risk for mothers who were not Hispanic white (i.e. black or other races), those with lower educational attainment, a history of adverse pregnancy outcomes, those who underwent Caesarian delivery, and those who delivered male infants, but these factors were not statistically significant at the 10% alpha level.

**Table 2:** Risk ratios (95% confidence intervals) for associations between preterm birth and covariates among PROTECT participants in fully-adjusted modified Poisson model^a^

Covariate	RR (95% CI)	*p*-value
Individual-level covariates		
Infant sex		
Female	1.00 (Ref)	
Male	1.18 (0.77, 1.81)	0.460
Mother’s age (years)	1.05 (1.00, 1.09)	0.035
Number of pregnancies, including this one		
This one only	1.00 (Ref)	
Have had other pregnancies	0.74 (0.42, 1.29)	0.282
Mother’s education		0.688
College	1.00 (Ref)	
High school or technical school	1.26 (0.73, 2.20)	
Less than high school	1.38 (0.56, 3.38)	
Marital status at first study visit		
Married	1.00 (Ref)	
Single, divorced, or cohabiting	1.78 (1.14, 2.78)	0.012
Employed at first study visit		
Yes	1.00 (Ref)	
No	1.66 (0.95, 2.89)	0.074
Family income per year, US dollars		
< 30,000	1.00 (Ref)	
≥ 30,000	0.93 (0.54, 1.58)	0.777
Mother’s race		
Hispanic white	1.00 (Ref)	
Black or other race	1.28 (0.84, 1.95)	0.247
History of adverse pregnancy outcomes		
No	1.00 (Ref)	
Yes	1.37 (0.79, 2.35)	0.258
Any cigarette smoke exposure in the home		
No	1.00 (Ref)	
Yes	1.01 (0.61, 1.67)	0.984
Alcohol consumption in past year, days per week		
0	1.00 (Ref)	
1 or more	0.70 (0.34, 1.45)	0.339
Delivery type		
Vaginal	1.00 (Ref)	
Caesarian	1.55 (0.99, 2.43)	0.057
Exercise habits		
Not exercise for > 30 min/day in past 3 months	1.00 (Ref)	
Exercised for > 30 min/day in past 3 months	0.94 (0.57, 1.57)	0.821
Maternal pre-pregnancy BMI		0.059
Normal or underweight	1.00 (Ref)	
Overweight	1.66 (1.02, 2.68)	
Obese	1.70 (1.00, 2.89)	
Municipal-level covariates		
Household income, 2013 inflation-adjusted US dollars		
Below median for PR (USD 22,754)	1.00 (ref)	
Above median	1.00 (1.00, 1.00)	0.343
Population density		
***≤*** median for PR (2760/mile^2^)	1.00 (ref)	
> median	1.00 (1.00, 1.00)	0.159
% adults (> 25 yr) with < high school education		
***≤*** median for PR (23.9%)	1.00 (ref)	
> median	1.07 (1.00, 1.14)	0.052
% occupied units with 1 or more severe housing problems^b^		
***≤*** median for PR (34.8%)	1.00 (ref)	
> Above median	0.99 (0.96, 1.03)	0.597
% non-white, non-Hispanic population		
***≤*** median for PR (35.7%)	1.00 (ref)	
> median	1.02 (0.99, 1.06)	0.252
Average unemployment rate, 2004–2013		
***≤*** median for PR (10.3%)	1.00 (ref)	
> median	1.07 (0.85, 1.35)	0.555
Age adjusted diabetes prevalence, 2004–2013		
***≤*** median for PR (12.4%)	1.00 (ref)	
> median	2.19 (1.00, 4.79)	0.051

^a^All models are adjusted for individual-level covariates (mother’s age, number of other children [alive or deceased], infant’s sex, gestational age, season of birth, education level, urban/rural residence, marital status, number of prenatal visits attended, and year of birth) and area/municipality-level covariates (including population density, income per capita, proportion of non-white non-Hispanic residents, average unemployment rate, proportion of occupied housing units with at least one of four severe US Department of Housing and Urban Development-designated defects, percentage of residents with a less than high school level of education, and the age adjusted prevalence of diabetes mellitus)

^**b**^These are designated by the US Department of Housing and Urban Development as overcrowding, lack of kitchen, lack of plumbing, and high housing cost

**Fig. 1:**
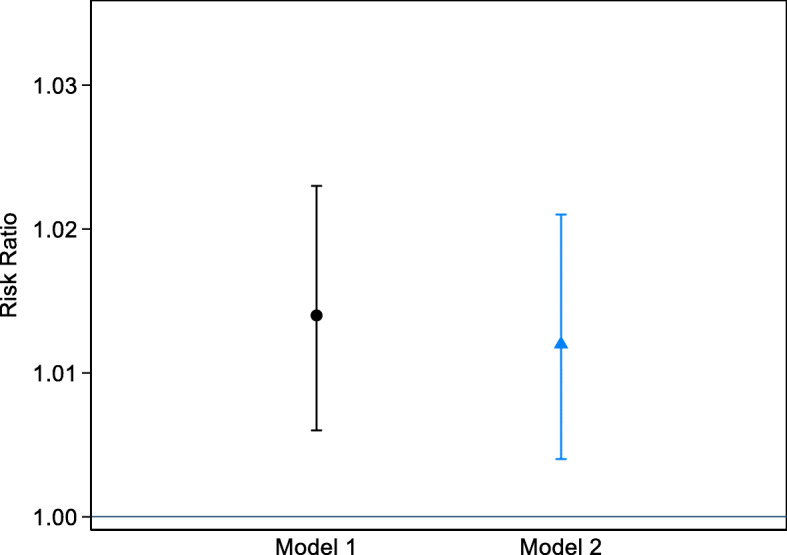
Risk ratios and 95% confidence intervals of preterm birth for an interquartile range increase in prenatal PM_2.5_ exposure among participants in the PROTECT cohort in Puerto Rico. Model 1: Adjusted for individual-level covariates, including mother’s age, number of other children (alive or deceased), infant’s sex, gestational age, season of birth, education level, urban/rural residence, marital status, number of prenatal visits attended, and year of birth. Model 2: Model 1 plus adjustment for area/municipality-level covariates, including population density, household income per capita, proportion of non-white non-Hispanic residents, average unemployment rate, proportion of occupied housing units with at least one of four severe US Department of Housing and Urban Development-designated defects, percentage of residents with a less than high school level of education, and the age adjusted prevalence of diabetes mellitus

We found that higher PTB risk was associated with an IQR increase in prenatal PM_2.5_ exposure, with similarly increased risks in models adjusting for individual level variables (RR 1.014; 95% CI 1.006, 1.023) and additionally for municipal-level covariates (RR 1.012, 95% CI 1.004, 1.021) (Fig. 1). We did not find evidence to suggest that the association between prenatal exposure to PM_2.5_ and risk of PTB varied by mother’s age, annual family income, adverse pregnancy history, pre-pregnancy body mass index, parity and education level, although there was a tendency toward marginally higher risk in the stratum of mothers who delivered female babies (Table 3). Excluding the mothers who reported having pre-eclampsia (19 who had full-term and 12 with preterm babies) did not alter the findings. When the analysis was restricted to only per-vaginal births, the results were also similar, albeit with a slightly reduced precision (RR 1.010, 95% CI 1.000, 1.024). Additionally, we did not observe statistically different PTB risks by pregnancy phthalate exposure levels, with interaction *p*-values of 0.55 and 0.86 for DBP and DiBP, respectively.

**Table 3:** Risk ratios and 95% CIs of preterm birth for an interquartile range increase in prenatal PM_2.5_ exposure among participants in the PROTECT cohort in Puerto Rico, stratified by mother’s characteristics^a^

Characteristic	Number of births (Number of PTBs)	Risk ratio (95% CI)	*p-*value
Age of mother (years)			0.28
< 25	539 (46)	1.01 (0.98, 1.03)	
≥ 25	553 (53)	1.01 (1.00, 1.02)	
Annual family income, US dollars			0.36
< 30,000	573 (62)	1.01 (0.99, 1.03)	
≥ 30,000	362 (26)	1.00 (0.99, 1.01)	
Infant sex			0.12
Male	572 (59)	1.00 (0.98, 1.02)	
Female	519 (40)	1.02 (1.00, 1.04)	
History of adverse pregnancy outcomes			0.72
No	833 (68)	1.00 (0.98, 1.03)	
Yes	259 (31)	1.01 (1.00, 1.02)	
Pre-pregnancy body mass index			0.74
Not obese	853 (68)	1.01 (1.00, 1.02)	
Obese	179 (21)	1.00 (0.97, 1.03)	
Number of pregnancies			0.98
Only this one	454 (39)	1.01 (0.99, 1.02)	
Have had other pregnancies	624 (59)	1.01 (0.99, 1.02)	
Education level			0.30
≤ High school	351 (47)	1.01 (0.98, 1.04)	
> High school	724 (51)	1.00 (0.99, 1.02)	
Prenatal DBP			0.50
Median (2.70) or less	354 (25)	0.83 (0.62, 1.10)	
More than median	352 (33)	0.97 (0.77, 1.24)	
Prenatal DiBP			0.63
Median (2.68) or less	355 (22)	0.94 (0.77, 1.15)	
More than median	351 (36)	0.97 (0.67, 1.39)	

*DBP* di-n-butyl phthalate, *DiBP* di-isobutyl phthalate

^a^ All estimates are from fully adjusted models, with adjustment for individual-level covariates (mother’s age, number of other children [alive or deceased], infant’s sex, gestational age, season of birth, education level, urban/rural residence, marital status, number of prenatal visits attended, and year of birth) and area/municipality-level covariates (including population density, income per capita, proportion of non-white non-Hispanic residents, average unemployment rate, proportion of occupied housing units with at least one of four severe US Department of Housing and Urban Development-designated defects, percentage of residents with a less than high school level of education, and the age adjusted prevalence of diabetes mellitus)

## Discussion

In a cohort of predominantly Hispanic and black women in Puerto Rico, average prenatal exposure to PM_2.5_ was associated with an increased risk of PTB. Higher maternal age and BMI, single marital status, and lower socioeconomic status were also associated with greater probability of PTB. This analysis benefitted from careful outcome ascertainment and a rich set of individual- and municipal-level covariates obtained in the PROTECT cohort. Most cohorts in which the role of PM_2.5_ in PTB has been evaluated have not included ethnic minority populations, despite consistent evidence of greater rates of adverse outcomes among black and Hispanic women [7, 28]. Our study adds to the comparatively thin evidence base for the impact of prenatal air pollutant exposure on PTB among non-white women. In particular, our results come from the first assessment of the PM_2.5_–PTB association in Puerto Rico, a setting with higher rates of PTB than most of the contiguous United States.

A recent systematic review identified 18 cohort studies that assessed the PM_2.5_–PTB association across the entire pregnancy period [13]. All were conducted in either mainland United States, Canada, China, Australia, the Netherlands, or England. Among the nine studies in which a positive association was observed, odds ratios ranged from 1.03 to 1.19 for an IQR change in PM_2.5_ exposure. In studies that did not find positive associations, the odds ratios were between 0.87 and 1.22 per IQR change, with confidence limits that included the null. In our Puerto Rican cohort, we estimated a risk ratio of 1.01. When we calculated a corresponding estimate of the odds ratio from our data, we obtained a value of 1.026, lower than reported in the subset of previous cohorts whose data were also consistent with an unambiguous positive association. This may be due in part to our analytical approach or our use of centrally-located EPA monitors to assign exposure, in the absence of finer spatiotemporal estimates for our study region. Notably, many of the US-based studies reported average exposure levels higher than we measured among our cohort, ranging from about 9.0 to 15.6 *μ* g/m^3^, which may also explain their reports of comparatively stronger associations [16, 32–34]. Several other differences likely underlie variations in estimated effect sizes across cohorts, including adequacy of confounder adjustment, study periods, study sites, and participant characteristics. While effect sizes may appear modest, the ubiquity of particulate matter make it an important risk factor – a recent estimate suggested that nearly one-fifth of all preterm births globally were linked to anthropogenic PM_2.5_ [35].

Few previous studies have assessed factors that may modify the association between PM_2.5_ and PTB. Some evidence suggests that the magnitude of risk may be higher among non-white women [36]. A recent systematic review reported that epidemiologic studies have demonstrated only weak evidence of modification by educational attainment, and that data on effect modification by income, occupation and area-level SES remain inconclusive [37]. In our analysis, we did not observe modification by various maternal characteristics, including age, income, BMI, and education level. Studies in the PROTECT cohort have demonstrated that pregnancy phthalate exposures (metabolites of DBP and DiBP) impact the risk of PTB [17]. No previous studies have reported whether the effect of PM_2.5_ on PTB varies by prenatal levels of these phthalates. In the PROTECT cohort, we did not find evidence of such PM_2.5_–phthalate interaction with respect to PTB outcomes. While it is plausible that some phthalates may result in increased inflammation and possibly make the fetus more susceptible to additional inflammatory stimulus from air pollutants, it is probable that the magnitude and range of pollutant exposures observed in our study population were too low to observed an interactive effect. In addition, low numbers have generally hampered efforts toward well-powered effect modification assessment in cohort studies [37], and our null findings require replication in additional studies.

Our data suggest that higher maternal age, lower socioeconomic status, higher BMI, and single marital status are associated with greater PTB risk, largely consistent with prior literature [28]. Women living in municipalities with higher prevalence rates of diabetes had increased risk. We also observed a non-statistically significant tendency toward higher PTB risk among women with a history of adverse pregnancy outcomes and women who were not Hispanic white (i.e. those belonging to black or other non-white ethnicities). The similarities between birth weight percentiles across term versus preterm infants may suggest that growth restriction may not have played a substantial role in biasing results in this cohort. While higher risk of PTB has been associated with strenuous work [28], the evidence regarding how self-motivated or leisure time physical activity correlates with preterm birth is more equivocal [38, 39], but suggests that physical activity may reduce the risk of PTB [40]. Exercise habits during pregnancy were not statistically associated with PTB risk in our cohort, although history of physical activity was self-reported, and thus may be measured with error.

The mechanisms through which exposure to particulate pollutants during pregnancy operates to potentially trigger PTB are not fully understood. Several avenues by which particulate matter may alter the timing of human parturition have been hypothesized, including oxidative stress, endocrine disruption, endothelial dysfunction, and hemodynamic imbalance [8, 10, 41]. Particulate matter may affect fetal development by altering blood viscosity and coagulability, which may impair the transfer of oxygen and nutrients across the placenta. Particulate matter has also been associated with pulmonary inflammation and placental inflammation, factors that may similarly interfere with normal transplacental oxygen and nutrient transport. Inflammation resulting from particulate matter could lead to endothelial dysfunction and higher risk of infection, which may result in preterm birth. Fine particulates may also increase blood pressure, which may induce hypertension, worsen existing hypertension, or cause pre-eclampsia. Fine particulate matter may be translocated via the placenta, possibly causing oxidative stress and DNA damage in the fetus, mother, or both. Finally, particulate matter may serve as an endocrine disruptor, interfering with fetal development and potentially causing preterm birth.

This study has limitations. Our exposure assessment relies on area-wide averages of measurements at EPA monitors, limiting the ability to take advantage of spatial variability in PM_2.5_ concentrations while also not accounting for maternal residential moves during pregnancy, since exposure assignment was based on municipality of residence at the time of birth. Both of these factors will contribute to exposure error, but such error will likely bias the estimated effects toward the null [42]. Additionally, the exposure contrast in our study area was relatively low, with PM_2.5_ concentrations ranging between 3 and 20 *μ* g/m^3^ over the duration of the pregnancies we analyzed. Also, as in most epidemiologic studies of air pollution, there remains the possibility of unmeasured confounding. However, we had access to many well-characterized individual and municipal-level potential confounders and effect modifiers, including phthalate exposures, which have not been studied before in the context of PM_2.5_ effects on PTB. The PROTECT cohort was restricted to women without major comorbidities, which means that this assessment of the PM_2.5_–PTB is not influenced by additional risk that may be associated with such morbidity, although it also implies that the results are generalizable only to a relatively healthy population. While we examined a prospectively followed cohort in a population for which no data on this association are available so far, a comparatively low number of preterm births led to imprecision around some of our estimates and reduced power for effect modification analyses.

## Conclusion

We observed an association between prenatal PM_2.5_ and PTB among Puerto Rican women who were free from major comorbid conditions. Older women, those with lower socioeconomic status, and those with BMI above 25 were at a higher risk for PTB. Exposure to even modest PM_2.5_ concentrations may contribute to adverse birth outcomes among relatively healthy women from ethnic minority populations.

## Data Availability

Data are available upon reasonable request to the authors and with institutional permission.
